# Student-Designed Cross-Sectional Pandemic Knowledge Survey of 8th−12th Grade Students, Milwaukee, WI, April 2020

**DOI:** 10.3389/fped.2021.622254

**Published:** 2021-06-11

**Authors:** Krish Vasudev, Hersh Singh, August A. Neumann, William M Zhao, Lyle Dominic Pelayo Mondano

**Affiliations:** ^1^Upper School Division, University School of Milwaukee, Milwaukee, WI, United States; ^2^Upper School Division, Marquette University High School, Milwaukee, WI, United States

**Keywords:** adolescent health, school health, COVID-19 survey, infectious disease, high school student

## Abstract

**Background:** The novel coronavirus and its effect on our society are unprecedented. Given the recent pandemic, numerous measures have been taken to protect our communities. We sought to understand our school community's knowledge and the measures that were taken by our school for our safety.

**Objective:** Our objective was to describe the overall understanding and attitudes of 8–12th grade students from a single institution during the initial phase of the Wisconsin's Governor's stay-at-home order.

**Methods:** A voluntary web-based survey was communicated to 8–12th grade students through their online school portal. Data were collected and analyzed using SurveyMonkey.

**Results:** There was a 20.2% response rate. Answers regarding the coronavirus, spread, and response to the coronavirus pandemic showed a high level of understanding of the virus and the actions necessary to prevent its spread.

**Conclusion:** Eight-twelfth grade students have a high level of understanding of the virus, its effects, and the safety measures implemented to protect society.

## Introduction

The Severe Acute Respiratory Syndrome-Related Coronavirus 2 (SARS-CoV-2) also known as the novel coronavirus is the virus behind the disease COVID-19 and the 2019–2020 pandemic, which has had a significant impact on the lives of many. SARS-CoV-2 is a virus that can cause severe respiratory damage to those infected and most testing positive have symptoms that range from none to mild. People can show signs of symptoms from 1 to 2 weeks after infection. Symptoms of the coronavirus can overlap with the common cold or the seasonal influenza virus, and can include fever, nausea, diarrhea, shortness of breath, headache, body aches, loss of smell or taste, fatigue, etc.^1^. Needless to say, this healthcare emergency is unprecedented in terms of how it has impacted our health and in terms of how it has prompted wide-scale social safety measures. To combat the spread of this virus, we stopped living out our daily routines and began living in a state of isolation to contain the spread, to decrease its morbidity, and to ensure that our healthcare system can handle the population that is infected. In Wisconsin, on March 23, 2020 there was a 102% increase in the number of positive cases in the preceding 72 h which led to the governor of Wisconsin to implement Emergency Order #12, Safer at Home Order^2^. During the onset of the pandemic and the Safer at Home Order, we chose to investigate the level of knowledge and attitudes regarding this novel virus, the pandemic, and the resulting dramatic changes implemented among 8th grade and high school students in a single educational institution in Wisconsin.

## Objective

The main objective of the study was to ascertain the level of knowledge that the University School of Milwaukee (USM) 8–12th grade student community had on the novel coronavirus. In addition to gaining new knowledge, we wanted to make the most of our quarantine to learn how we could protect ourselves and others, and the procedures needed to combat the virus effectively. Another main reason for conducting this survey was to learn more about aspects of the coronavirus that need to be addressed more urgently and to see what information about the coronavirus should be made more public. We sensed that this would be very beneficial to help address this pandemic and keep infection rates as low as possible.

## Methods

An electronic survey of 21 questions was created by a team of five 8th grade students using the Survey Monkey web application (San Mateo, California; http://www.surveymonkey.com)^3^. for 8th to 12th-grade students who attend University School of Milwaukee, River Hills, WI. The survey was reviewed by school administrators for level of comprehension. Between April 3–28, 2020, 78 8th grade students and 421 high school students were provided a link to the survey through their school portal. The survey was anonymous with the option to include one's name if the individual was interested in participating in a drawing for a school-themed prize. Five randomly selected participants received gifts for participating in the survey. A copy of the survey can be found in the Supplementary Material.

## Results

A total of 101 out of 499 students responded, giving a response rate of 20.2%. Regarding knowledge of the virus, 95.05% of the respondents correctly answered a question on how the novel coronavirus is spread/transmitted between people, 100% knew symptoms at the time of the survey, 90.1% were aware that a few virions could spread disease, 96.04% identified the lung as the most commonly affected organ, 90.1% identified 1–14 day latency period before the onset of symptoms, 92.08% identified the 60+ age group as being high risk for disease, and 83.17% identified COVID-19 as more contagious than the seasonal influenza virus.

Most students were also aware of the measures taken to prevent the spread, with 99.01% identifying ways to prevent the spread of SARS-CoV-2. In addition, 91.09% identified that most patients recover from infection. All students (100%) were aware of the term “social distancing” at the time of the study, while 73.27% reported that the pandemic changed their hygiene habits.

The study was initiated during the Wisconsin state-mandated safer-at-home order (see text footnote 3) in April of 2020 and respondents were asked if they engaged in any form of social gathering in the 2 weeks prior to the initiation of the order. Of the study subjects, 23.76% engaged in playdates/sleepovers, 13.86% attended a party, 10.89% attended a large family dinner, 17.82% traveled out of state, 58.42% engaged in none of these activities.

The respondents were then asked about their understanding and attitude toward the safer-at-home order and what was considered an essential worker. Of the student population, 21% correctly identified all the occupations of essential workers, 51.49% of respondents had parents who were identified as essential workers, 98.02% were supportive of the decision to close schools, 89.11% were optimistic that the government, community, and financial outlook would survive the pandemic, and 99.01% had discussions regarding the pandemic with their families.

The University School of Milwaukee utilizes an online learning platform during the routine school year, and this platform facilitated the transition to online learning. Students were asked if they felt that online schooling was sufficient for their education, of which 78.22% of students were satisfied, and 97.03% reported that they engaged in other virtual learning platforms during the period of school closure.

A summary of questions and the respondent's answers can be found in Table 1.

**Table 1:** Pandemic knowledge survey responses of 8th–12th students.

**Questions**	**% correct**	**% incorrect**
How is the novel coronavirus (SARS-CoV-2) spread/transmitted between people?	95.05	4.95
What are the common initial symptoms of infection by this virus?	100	0
Which of the following statements is true?	90.1	9.9
The organ that is initially most commonly affected by the coronavirus, SARS-CoV-2 is:	96.04	3.96
What is the time between infection and development of symptoms (incubation period) due to SARS-CoV-2?	90.10	10
Which age group is most severely affected by the disease COVID-19?	92.08	7.92
SARS-CoV-2 is more contagious and harmful compared to the seasonal flu/influenza virus.	83.17	17
What are ways that we can prevent the spread of SARS-CoV-2?	99.01	1
There is a vaccine available for SARS-CoV-2 that causes COVID-19.	97.03	3
Even without any specific treatment, most patients who get infected with SARS-CoV-2 recover.	91.09	8.91
The following questions are answered Yes or No	**Yes (%)**	**No (%)**
Are you aware of the term social distancing?	100	0
During the 2 weeks before Governor Evers issued the safer at home order effective March 25th, 2020, did you or members of your family engage in social gatherings such as (click all that apply)^a^:	See below	
Which of the following constitutes an essential worker during the Stay-at-Home order? Select all that apply^b^.	See below	
Have your parents or guardians had to work and provide essential services during the stay at home order issued by Governor Evers?	51.49%	48.51%
Do you feel that our school, state and federal governments made a wise decision to close the schools to prevent the spread of this infection?	98.02%	1.98%
Are you optimistic that with the support from the government and our community, our community, and financial outlook will survive this pandemic?	89.11%	10.89%
Has the SARS-CoV-2 outbreak changed your hygiene habits (e.g., how often and how you wash your hands)?	73.27%	26.73%
Have your parents/family members/caretakers talked to you about COVID-19?	99.01%	0.99%
During this period of school closure, have you engaged in any form of virtual learning (Skype lessons, lessons via FaceTime, online learning for out of school activities)?	97.03%	2.97%
Does online learning provide enough resources for your education?	78.22%	21.78%

a *58.42% did not engage in activities listed, 24.76% participated in playdates and/or sleepovers, 13.86% attended parties, 10.89% large family dinners, 28.71% had visitors in home, and 17.82% traveled out of state*.

b *95.05% answered health and social care, 30.69% education and childcare, 92.08% food and other necessary goods, 67.33% key public services, 72.28% local and national government, 65.35% utility workers, 82.18% public safety and national security, 48.51% transport. 21% of respondents chose all of the right answers listed*.

## Discussion

The coronavirus pandemic affected all aspects of everyday life and has redefined normality. While our understanding of this pandemic changes daily, our survey shows that basic understanding regarding the transmission of the virus, the disease it causes, and the recovery process among 8th graders and high schoolers surveyed is robust. This has also underlined the importance of technology in our current day society where more and more of our interactions are moving to virtual platforms in the areas of education, employment, group gatherings, and even patient care.

The limitations of our study stem from inclusion of students from a single independent school located in the suburb of a mid-size city and where the majority of the students identified parents or guardians who provided essential services at the onset of the pandemic. Reponses can be affected by the geographic location of the school and its students given that the pandemic had geographically disproportionately affected students, their families, and schools. The results of our study could be generalizable to students from similar schools and background. Information related to the pandemic was widely disseminated through education in schools, household conversations, news, and social media. Therefore, the knowledge related to COVID-19 among students in our age group across the country may be similar irrespective of the single school cross sectional nature of this study.

To our knowledge, this is the first survey conducted to assess knowledge and attitudes toward the novel coronavirus pandemic in 8th grade and high school students in Wisconsin. In our study, we found that the students had a strong understanding of this virus, its effects, and the changes implemented to protect society. We have had to navigate uncharted territories regarding all of our lives, in school and out of school. We are grateful to our school community for being flexible and supportive in their approach to the pandemic. Our generation will be resilient and be remembered for what we faced and learned during this historic time.

## Implications for Health Behavior or Policy

The implications of COVID-19 are unprecedented. According to the UNESCO website, “over 1.5 billion learners in 165 countries are affected by COVID-19 school closures”^4^. The magnitude of students affected by the current pandemic is unlike any other health emergency experienced in our lifetime. We understand that schools are facing multiple decisions on how to make the school environment safe, while maintaining the quality of education looking forward to the next school year^5^. We had a good understanding of the coronavirus, how it is diagnosed, and support the decisions to close schools in the spring of 2020. Students were able to correctly answer general questions on the pandemic and protective measures that are needed to “flatten the curve.” The understanding of this disease by the students is likely to have a protective effect in teenagers' risk of infection. We look forward to the ongoing educational opportunities which will arise moving forward. Policy makers should know that students can understand the harmful effects this virus can have on our health and on the health of the people around us. Students are resilient and can easily adapt to modified school policies to ensure our safety and of those around us.

The pandemic has also taught all of us some important lessons. It has brought to the forefront human vulnerability and made us realize that mother nature still rules. While there has been a significant toll in terms of human life and the negative impact on the economy, the pandemic has also had a positive impact in some respects. We are beginning to realize the value of everyday things that we used to take for granted. It has exposed the distinct differences between “wants” and “needs” and shown us what is essential in life. It has also brought us together as communities as we realize the importance of human contact when forced into isolation. Overall, we believe that we will emerge from this stronger and more resilient.

## Data Availability Statement

The original contributions presented in the study are included in the article/Supplementary Material, further inquiries can be directed to the corresponding author/s.

## Ethics Statement

Ethical review and approval was not required for the study on human participants in accordance with the local legislation and institutional requirements. Written informed consent from the participants' legal guardian/next of kin was not required to participate in this study in accordance with the national legislation and the institutional requirements.

## Author Contributions

All authors contributed in the design of the study and in the creation and editing of the manuscript.

## Conflict of Interest

The authors declare that the research was conducted in the absence of any commercial or financial relationships that could be construed as a potential conflict of interest.

## Footnotes

^1^https://www.cdc.gov/coronavirus/2019-nCoV/index.html (accessed March 24, 2020).

^2^https://www.surveymonkey.com/results/SM-K2VRFYNX7/

^3^https://evers.wi.gov/Documents/COVID19/EMO12-SaferAtHome.pdf (accessed March 24, 2020).

^4^https://en.unesco.org/news/unesco-rallies-international-organizations-civil-society-andprivate-sector-partners-broad (accessed July 5, 2020).

^5^https://dpi.wi.gov/sites/default/files/imce/sspw/pdf/Education_Forward_web.pdf (accessed May 28, 2020).

