# Supplementary material for: Student-Designed Cross-Sectional Pandemic Knowledge Survey of 8th−12th Grade Students, Milwaukee, WI, April 2020
Source: Front Pediatr. 2021 Jun 11;9:622254. doi: 10.3389/fped.2021.622254 (PMC8225947; doi:10.3389/fped.2021.622254)
Supplement: Supplementary file 1 [file Data_Sheet_1.PDF]

We Value Your Time. Our understanding of the SARS-CoV-2 and COVID-19 pandemic.

We are living in a time where a new disease called COVID-19 has become a public health emergency. This disease is caused by Severe Acute Respiratory Syndrome-Related Coronavirus 2 (SARS-CoV-2), also known as the novel coronavirus. This health emergency is unprecedented in its effects on our health and how our society has responded to protect us. We have created this survey to evaluate the medical awareness and our views of this virus, disease, and ways to protect ourselves during this time. We thank you in advance and value your time and input. Five randomly selected participants will receive a USM related gift. The results of this survey will be published at a future date.

1. How is the novel coronavirus (SARS-Cov 2) spread/transmitted between people?

- ◯ Through the air by coughing and sneezing (droplet infection)
- ◯ Close personal contact, such as touching or shaking hands
- ◯ Touching an object or surface with the virus on it, then touching your mouth, nose or eyes before washing your hands
- ◯ In rare cases, fecal contamination (not washing your hands after going the bathroom)
- ◯ All the above

2. What are the common initial symptoms of infection by this virus?

- ◯ Loss of smell, fever, dry cough, difficulty breathing
- ◯ Chest pain and swelling of the legs
- ◯ Loss of vision and headaches
- ◯ Rash all over the body

3. Which of the following statements is true?

- ◯ One needs to be infected by millions of coronavirus particles/virions to cause COVID-19
- ◯ Only a few coronavirus particles/virions are enough to cause COVID-19

4. The organ that is initially most commonly affected by the coronavirus, SARS-CoV-2 is:

- ◯ Skin
- ◯ Heart
- ◯ Stomach
- ◯ Lungs
- ◯ Brain

5. What is the time between infection and development of symptoms (incubation period) due to SARS-Cov 2?

- ◯ 1-14 days
- ◯ 15-21 days
- ◯ 22-28 days

6. Which age group is most severely affected by the disease COVID-19?

- ◯ 1-5 years
- ◯ 6-20 years
- ◯ 21-40 years
- ◯ 41-60 years
- ◯ 60+ years

7. SARS-Cov 2 is more contagious and harmful compared to the seasonal flu/influenza virus.

- ◯ True
- ◯ False

8. What are ways that we can prevent the spread of SARS-Cov 2?

- ◯ Washing hands with soap and water for 20 seconds
- ◯ Staying home to prevent the spread of this viral illness
- ◯ Avoiding sick contacts
- ◯ All of the above

9. There is a vaccine available for SARS-CoV-2 that causes COVID-19.

- ◯ True
- ◯ False

10. Even without any specific treatment, most patients who get infected with SARS-Cov 2 recover.

- ◯ True
- ◯ False

11. Are you aware of the term social distancing?

- ◯ True
- ◯ False

12. During the two weeks before Governor Evers issued the safer at home order effective March 25th, 2020, did you or members of your family engage in social gatherings such as (click all that apply):

- [ ] Playdates and/or sleepovers
- [ ] Visitors in your home
- [ ] Parties
- [ ] Travel out of state
- [ ] Large family dinners
- [ ] None of the above

13. Which of the following constitutes an essential worker during the Stay-at-Home order? Select all that apply.

- [ ] Health and social care
- [ ] Local and national government
- [ ] Education and childcare
- [ ] Utility workers
- [ ] Food and other necessary goods
- [ ] Public safety and national security
- [ ] Key public services
- [ ] Transport

14. Have your parents or guardians had to work and provide essential services during the stay at home order issued by Governor Evers?

- ○ Yes
- ○ No

15. Do you feel that our school, state and federal governments made a wise decision to close the schools to prevent the spread of this infection?

- ○ Yes
- ○ No

16. Are you optimistic that with the support from the government and our community, our community and financial outlook will survive this pandemic?

- ○ Yes
- ○ No

17. Has the SARS-Cov 2 outbreak changed your hygiene habits (e.g. how often and how you wash your hands)?

- ○ Yes
- ○ No

18. Have your parents/family members/caretakers talked to you about COVID-19?

- ○ Yes
- ○ No

19. During this period of school closure, have you engaged in any form of virtual learning (skype lessons, lessons via facetime, online learning for out of school activities)?

- ◯ Yes
- ◯ No

20. Does online learning provide enough resources for your education?

- ◯ Yes
- ◯ No

21. Optional. Please include your name to be included in the drawing.
